# What Is the Best Proximal Anastomosis for the Free Right Internal Thoracic Artery during Bilateral Internal Thoracic Artery Revascularization? A Prospective, Randomized Study

**DOI:** 10.1155/2014/972832

**Published:** 2014-02-06

**Authors:** S. Neragi-Miandoab, R. E. Michler, F. Lalezarzadeh, R. Bello, J. J. DeRose

**Affiliations:** Department of Cardiothoracic Surgery, Montefiore Medical Center, Albert Einstein College of Medicine, Bronx, NY 10467, USA

## Abstract

*Objective*. Bilateral internal thoracic artery (BITA) grafting provides improved graft patency and potential survival advantage in selected patients as compared to single left internal thoracic artery (LITA) revascularization. The ideal functional BITA configuration remains controversial. *Methods*. Patients undergoing planned BITA revascularization with greater than 75% stenosis in both the left anterior descending artery (LAD) and in a circumflex branch were prospectively randomized to one of two proximal free right internal thoracic artery (RITA) connections directly off the aorta (Ao) (*n* = 12) or as a “t” graft off the LITA (t) (*n* = 12). The LITA was placed to the LAD in all cases, and the RITA was placed to a single lateral wall vessel. Intraoperative transit time flow measurements of all arterial grafts were performed, and RITA fractional flow parameters were compared between the 2 groups. *Results*. There were no differences in preoperative patient variables between the two groups. Cross-clamp times (91.5 + 15.3 versus 68.0 + 12.5 minutes, *P* < 0.01) and total cardiopulmonary bypass times (109.0 + 16.2 versus 85.0 + 15.1 minutes, *P* < 0.01) were shorter in the t group. The Ao group demonstrated significantly higher mean RITA flow (38.3 ± 13.5 versus 22.1 ± 9.5, *P* < 0.01), mean RITA conductance (flow/mean arterial pressure) (0.45 ± 0.16 versus 0.28 ± 0.11, *P* < 0.01), RITA fractional flow (0.52 ± 0.15 versus 0.36 ± 0.11, *P* < 0.01), and RITA fractional conductance (0.51 ± 0.15 versus 0.36 ± 0.11, *P* < 0.01) than the “t” grafted patients. Thirty-day mortality and wound infection were 0% for each group. Over an average of 42.8 + 6.6 months of followup there were no mortalities in either group. Repeat angiography were performed in 4 patients (33%) in the Ao group and 2 patients in the t group (16%). One occluded RITA graft and one ostial RITA stenosis were detected in the Ao group. *Conclusions*. Acute flow measurements indicate that the free RITA anastomosed to the aorta provides more acute fractional RITA flow than composite “t” grafting to the LITA. Longer-term angiographic and clinical followup are necessary to determine the consequences of these acute hemodynamic findings.

## 1. Introduction

Bilateral internal thoracic artery (BITA) grafting has been shown to improve survival and decrease the need for reintervention and reoperation over long-term followup when compared to single internal thoracic artery grafting [1]. However, the optimal technical configuration for the use of BITAs still remains controversial. The right internal thoracic artery (RITA) can be used as a pedicled graft, as a free graft sewn to the aorta (or vein hood), or as part of a pedicled “y” or “t” graft in which the free RITA is sewn to the pedicled LITA in an end-to-side fashion. All three arrangements have their advantages and disadvantages. The pedicled RITA has the advantage of keeping its proximal connection undisturbed but suffers from a lack of length to reach all left-sided targets comfortably [2]. The free RITA can be used with adequate length to most circumflex targets but the proximal aortic-RITA connection does offer some technical challenges which can affect long-term patency [3].

The “t” or “y” connection allows for the most length and complete left-sided revascularization, but some surgeons are reticent to base the entire revascularization strategy on the LITA inflow [4]. Retrospective studies have not been able to demonstrate differences in patency between in situ and “t” graft configurations [1, 4, 5]. There is a paucity of data on the use of the free RITA off the aorta compared to these two other configurations. The aim of the present study was to define the acute hemodynamics of the two most versatile BITA configurations (“t” grafting and free RITA aortic grafting) in a prospective, randomized fashion.

## 2. Patients and Methods

### 2.1. Study Design

All patients referred to a single surgeon for isolated coronary artery bypass grafting from January 2008 to October 2009 were screened according to the inclusion and exclusion criteria listed in Table 1. A computerized random number generator was used to assign patients to one of two surgical strategies: BITA “t” grafting (t) or free RITA grafting off the aorta (Ao).

Randomization occurred in a 1 : 1 fashion in the operating room following the sternotomy. The LITA was placed to the LAD, and the RITA was placed to the largest lateral wall vessel in all cases. The cross-clamp time was naturally longer in off-aorta cases, since we had to perform an additional proximal anastomosis while the aorta was cross-clamped. The extended cross-clamp time resulted in longer bypass time. The LITA-RITA “t” graft was constructed prior to going on cardiopulmonary bypass. All off-aorta RITAs were anastomosed to the vein hood of the vein grafts. A completely direct anastomosis of the RITA off the aorta was not performed in any of the cases. No sequential grafting was performed in order to provide equipoise between the anatomic outflows of both revascularization techniques. All other grafts were performed with saphenous veins. The decision to perform the operation off pump or on pump was left to the surgeon's discretion. All patients gave written signed consent to participate in the study, and the study protocol was approved by the Institutional Review Board at our institution.

Postoperative hospital outcomes were obtained prospectively from our institutional database which is used to populate both the Society of Thoracic Surgeons database and the New York State database. Long-term mortality was evaluated with the Social Security Death Index.

Subsequent coronary angiograms performed at our institution were reviewed in followup when available.

### 2.2. Flow Measurement Studies

Following the construction of all bypass grafts and upon separating from cardiopulmonary bypass, duplex Doppler transit time flow measurements were made of all grafts (Medi-Stim, Norway). Stable hemodynamics were obtained and measurements were performed at a mean arterial blood pressure of 70–80 mmHg. Data from the Medi-Stim was recorded at 95–100% probe contact as measured by the device. Simultaneous continuous blood pressure, electrocardiogram, and flow measurements were also recorded. Mean flow was recorded and coupled with the ECG tracing to be correctly differentiated from systolic and diastolic flow. The diastolic filling (DF%), defined as the blood volume filling in diastole divided by the total blood volume in one heart cycle, was calculated by the Medi-Stim. Similarly, patency was confirmed with each graft by measuring the pulsatility index (PI) as defined by the following equation: PI = (Max flow − Min flow)/Mean Flow Volume.

We sought to define the RITA fractional flow for each BITA configuration. This was defined by the following equation: RITA fractional flow = RITA flow/(RITA flow + LITA flow). In order to correct for slight differences in blood pressure during each flow measurement, we calculated a conductance for each instantaneous flow measurement defined as mean flow/mean arterial blood pressure. The fractional RITA conductance was then defined as RITA conductance/(LIMA conductance + RITA conductance).

### 2.3. Patients

From January 2008 to October 2009, 24 patients met inclusion criteria and were enrolled into the study. The patient characteristics are listed in Table 2. Randomization matched the two groups well. There were slightly more cases performed off pump in the t group (33% versus 8.3%); however, this did not reach statistical significance. Of those cases performed on pump, the cross-clamp (*P* < 0.01) and the cardiopulmonary bypass time (*P* < 0.01) were statistically lower in the t group than the Ao group.

### 2.4. Statistical Analysis

Data was analyzed using SPSS software (version 11.5). Categorical variables are reported as percentages and continuous variables are reported as mean + SD, and 95% confidence interval is reported. Categorical variables were analyzed using Chi square test and continuous variables were analyzed using independent sample 2 tailed *t*-test.

## 3. Results

The intraoperative flow measurements and calculations are listed in Table 3. The integrity of the bypass grafts in both groups was similar as no statistically significant difference in LITA or RITA pulsatility indices or diastolic filling percentage was detected among the two groups. The RITA mean flow was significantly higher in the Ao group than in the t group. When corrected for instantaneous mean arterial blood pressure with a conductance measurement (flow/mean pressure), there remained a significant increase in the Ao group. However, the LITA flow and conductance between both groups remained equal. The fractional RITA flow was significantly higher in the Ao group, and this remained statistically significant when the RITA fractional conductance was compared.

The postoperative outcomes were excellent in both groups (Table 4). There were no wound infections in either group despite a high incidence of diabetes mellitus in both the Ao and t groups. One patient in the t group with a preoperative creatinine of 3.5 required postoperative hemodialysis. Thirty-day mortality was 0%, and all patients are alive at a mean followup of 42.8 + 6.6 months (range 31–53 months).

Postoperative cardiac catheterization was performed at the discretion of the patient's physicians based on symptoms or the results of noninvasive testing (Table 5). Four patients in the Ao group underwent cardiac catheterization at a mean duration of 12.5 ± 3.3 months from surgery. Two patients in the t group underwent cardiac catheterization at a mean duration of 20.0 ± 8.5 months from surgery. All LITA grafts among the 6 patients were patent. Among the Ao patients there was one occluded RITA graft and one proximal RITA stenosis which was treated with percutaneous coronary intervention. Both RITA grafts in the t group were patent without stenosis.

## 4. Discussion

The RITA has been shown to be a durable and consistent conduit when used as a second arterial graft together with LITA-LAD grafting [6–8]. Most previous investigations have evaluated the difference between in situ RITA grafting and free RITA grafting as part of composite “t”-graft configuration. In a 5-year followup, Hwang and colleagues demonstrated no significant difference in RITA graft patency on early, 1-year and 5-year postoperative angiograms between in situ and “t” graft configurations [4]. Similarly, in a prospective, randomized study of 304 patients, Glineur and colleagues found no angiographic differences at 6 months between in situ RITA grafting and composite “y” RITA grafting [9].

However, few prospective studies exist comparing the RITA proximal connection either to the aorta or to the LITA. Proponents of free RITA grafting off the aorta have argued that a potential steal phenomenon can occur when the LITA and RITA are sharing a single inflow trunk [10].

Although intraoperative studies have suggested that the flow reserve is adequate with a composite “t” configuration [2, 11, 12], conflicting results regarding flow reserve in the proximal LITA trunk as measured by postoperative Doppler have been reported [10]. Some authors have raised concern that a “t” configuration may be associated with steal phenomenon in times when increased blood flow is required in myocardium [10]. Measuring the fractional flow resistance in both branches of a BITA “y” composite graft, Glineur et al. [9] did not observe a steal phenomenon in the RITA from the LITA. Alternatively, proponents of composite “t” grafting have argued that an aortic-RITA proximal connection is associated with both technical issues and flow mismatch concerns which can compromise graft patency [13].

Calafiore and colleagues [14] reported a lower patency for free RITA grafts proximally anastomosed to the aorta as opposed to those sewn to the LITA. Gaudino et al. showed similar results in a followup to these data [13]. However, Fukui et al. showed no difference in 1-year angiographic patency rate among patients with composite RITA grafting (89.8%) and 10 patients undergoing aortic grafting (90%) [15]. The present, prospective randomized study reveals that there is greater absolute and fractional flow in the RITA when it is proximally anastomosed to the aorta compared to a composite graft configuration. Similarly, the acute hemodynamic measurements made in our study support the fact that there is no significant steal phenomenon associated with a composite BITA arrangement as the LITA flow remained equal between the t and Ao groups. Nonetheless, the consequences of this increased flow provided by an aortic-RITA connection remain unclear. Our study was not designed as an intermediate or long-term angiographic followup of these two BITA revascularization strategies. However, the 6 patients who underwent clinically directed postoperative angiography at 10 to 26 months provide supportive data for previous conclusions.

Those patients not undergoing angiography were all symptom free with normal postoperative nuclear stress testing. If it is assumed that this cohort represents patent arterial grafts, then the overall 3-year RITA patency is 100% in our t group and 89% in our group. These patency numbers are consistent with previous RITA patency reports. It is impossible to make significant assumptions on the basis of the small number of postoperative angiograms. However, it is interesting that this study documented higher RITA flow acutely, and in 3–5-year followup there were 2 aortic-RITA grafts that developed late problems (one occlusion and one proximal stenosis). Although we do not have early angiography on these two patients, it can be assumed that adequate Medi-Stim flow parameters confirmed immediate intraoperative patency. The technical pitfalls which may have an impact on anastomosis and its patency rate include the thickness of the aorta, the size of the RITA, the fragility of both LITA and RITA, and surgeon's experience. Some of these factors may require a change in strategy per surgeon's discretion. In conclusion, the present prospective, randomized study confirms that there are greater acute flow parameters in the RITA when it is sewn proximally to the aorta than when it is used as a composite graft to the LITA. The small, clinical angiographic followup of these patients suggests that there may be a higher incidence of restenosis in patients undergoing proximal aortic anastomosis. A larger, prospective randomized study with planned angiographic followup will be necessary to definitively answer this question.

## Figures and Tables

**Table 1:** Inclusion and exclusion criteria.

Inclusion criteria	Exclusion criteria
Angiographic evidence of >75% stenosis in LAD and lateral wall CA	Emergency surgery
Isolated CABG	LVEF < 25%
Availability of both RITA and LITA	Renal failure on HD
Nonemergent procedure	Surgical plan for sequential BITA grafting
Age < 80	Uncontrolled DM (preoperative fasting glucose >400 mg/dL)
Ability to understand and consent to the procedure	Inability to provide informed consent

**Table 2:** Preoperative and intraoperative patient characteristics.

	Ao	t	*P*
Age (years)	62.0 ± 6.0	66.3 ± 7.5	NS
Male	9 (75%)	10 (83%)	NS
LVEF (%)	53.0 ± 10.7	56.3 ± 13.8	NS
Preoperative creatinine (mg/dL)	1.0 ± 0.8	1.1 ± 0.3	NS
COPD	1 (8%)	3 (25%)	NS
Diabetes	5 (42%)	6 (50%)	NS
Cerebrovascular disease	1 (8%)	1 (8%)	NS
Prior stroke	1 (8%)	0	NS
Peripheral vascular disease	1 (8%)	0	NS
NY State risk score	0.01 ± 0.01	0.01 ± 0.005	NS
Off pump	1 (8%)	4 (33%)	NS
Cross-clamp time (minutes)	91.5 ± 15.3	68.0 ± 12.5	<0.01
Bypass time (minutes)	109.0 ± 16.2	85.0 ± 15.1	<0.01
Number of distal anastomoses	3.2 ± 0.6	3.2 ± 0.6	NS

LVEF: left ventricular ejection fraction; COPD: chronic obstructive pulmonary disease.

**Table 3:** Intraoperative flow measurements.

	Ao	t	*P*
LITA mean flow	37.3 ± 18	41.2 ± 16.0	NS
RITA mean flow	38.3 ± 13.5	22.1 ± 9.5	<0.01
LITA pulsatility index	2.4 ± 0.7	3.1 ± 1.2	NS
RITA pulsatility index	2.8 ± 1.4	2.5 ± 0.9	NS
LITA diastolic filling	74.8 ± 5.2	68.5 ± 10.3	NS
RITA diastolic filling	63.0 ± 10.9	65.9 ± 8.6	NS
LITA conductance	0.44 ± 0.22	0.52 ± 0.19	NS
RITA conductance	0.45 ± 0.16	0.28 ± 0.11	<0.01
RITA fractional flow	0.52 ± 0.15	0.36 ± 0.11	<0.01
RITA fractional conductance	0.51 ± 0.15	0.36 ± 0.11	<0.01

Conductance: flow/mean arterial blood pressure.

**Table 4:** Postoperative outcomes.

	Ao	t	*P*
Sternal wound infection	0	0	NS
Post-op stroke	0	0	NS
Post-op pneumonia	0	1 (8%)	NS
Prolonged ventilation	0	0	NS
Post-op atrial fibrillation	5 (42%)	4 (33%)	NS
Disposition home	12 (100%)	11 (92%)	NS
Disposition nursing facility	0	1 (8%)	NS
30-day mortality	0	0	NS
Post-op renal failure	1 (8%)	0	NS
LOS (days)	6.0 ± 2.0	6.0 ± 1.6	NS

**Table 5:** Follow-up angiography.

	Ao	t
*n*	4 (33%)	2 (16%)
Time to catheterization (months)	12.5 ± 3.3	20.0 ± 8.5 months
LIMA patency	100%	100%
RIMA patency	11 (92%)	100%
RIMA stenosis	1 (9%)	0
